# Skinfolds in nulliparous women and their association with pregnancy complications: a multicenter cohort study in Brazil

**DOI:** 10.61622/rbgo/2026rbgo31

**Published:** 2026-10-30

**Authors:** Marta Pedro Nhauche, Maria Julia Miele, Jussara Mayrink, Rafael Bessa de Freitas Galvão, Maria Laura Costa, Francisco Edson de Lucena Feitosa, Edilberto Alves Pereira da Rocha, Débora Farias Leite, Ricardo Porto Tedesco, Danielly Scarenello Nunes Santana, Karayna Gil Fernandes, Anic Campos Alves, Janete Vettorazzi, Iracema Calderon, Renato Passini, João Paulo Souza, José Guilherme Cecatti, Renato Teixeira Souza

**Affiliations:** 1 Universidade Estadual de Campinas Faculdade de Ciências Médicas Departamento de Ginecologia e Obstetrícia Campinas SP Brazil Departamento de Ginecologia e Obstetrícia, Faculdade de Ciências Médicas, Universidade Estadual de Campinas, Campinas, SP, Brazil.; 2 Universidade Federal do Ceará Maternidade Escola Assis Chateaubriand Fortaleza CE Brazil Maternidade Escola Assis Chateaubriand, Universidade Federal do Ceará, in Fortaleza, CE, Brazil.; 3 Universidade Federal do Pernambuco Faculdade de Ciências Médicas Departamento de Ginecologia e Obstetrícia Recife PE Brazil Departamento de Ginecologia e Obstetrícia, Faculdade de Ciências Médicas, Universidade Federal do Pernambuco, Recife, PE, Brazil.; 4 Faculdade de Medicina de Jundiaí Departamento de Ginecologia e Obstetrícia Jundiaí SP Brazil Departamento de Ginecologia e Obstetrícia, Faculdade de Medicina de Jundiaí, Jundiaí, SP, Brazil.; 5 Universidade Federal do Rio Grande do Sul Maternidade do Hospital de Clínicas Departamento de Ginecologia e Obstetrícia Porto Alegre RS Brazil Departamento de Ginecologia e Obstetrícia, Maternidade do Hospital de Clínicas, Universidade Federal do Rio Grande do Sul, Porto Alegre, RS, Brazil.; 6 Universidade Estadual Paulista Faculdade de Medicina de Botucatu Departamento de Ginecologia e Obstetrícia Botucatu Brazil Departamento de Ginecologia e Obstetrícia, Faculdade de Medicina de Botucatu, Universidade Estadual Paulista, Botucatu, Brazil.; 7 Universidade de São Paulo Faculdade de Medicina de Ribeirão Preto Departamento de Medicina Social Ribeirão Preto SP Brazil Departamento de Medicina Social, Faculdade de Medicina de Ribeirão Preto, Universidade de São Paulo, Ribeirão Preto, SP, Brazil.

**Keywords:** Skinfold thickness, Nutritional status, Pregnant women, Pregnancy complications

## Abstract

**Objective:**

This study aims to determine the trajectory of the sum of skinfolds in nulliparous women and its association with pregnancy complications.

**Methods:**

We used data from two multicenter longitudinal prospective cohort studies conducted in Brazil. These cohorts followed nulliparous pregnant women at usual risk, with no previous history of severe disease, starting between 19 and 21 weeks of gestation. Data from women who participated in the first visit of the study, women with data duly filled out in the SAMBA and MAES databases, were included in the study. The characterization of skinfolds was based on the quartiles established at the 1st study visit. The χ^2^ test was applied to compare sociodemographic differences for categorical variables, and for numerical variables, the Mann-Whitney test and the Shapiro-Wilk normality test were applied.

**Results:**

In total, 1,527 nulliparous women were considered in the analysis. There was an increase of all skinfolds over the three study visits. The variation of skinfolds throughout pregnancy differed according to maternal characteristics such as BMI, maternal age and income. Only women with no major complication showed an increase of the sum of the skinfold in the second study visit regardless the maternal BMI. Obese and overweight women who presented preeclampsia increased the sum of skinfolds between the first and the second visits.

**Conclusion:**

Our study suggest that the trajectory of skinfolds differ according to maternal characteristics, especially maternal BMI and monitoring their change over pregnancy may be a resourceful tool in identifying women who may develop pregnancy complications.

## Introduction

Nutritional status is a determining factor in the health of women and fetuses, as it influences fetal development and growth, as well as the evolution of pregnancy.^(1,2)^ Women in the underweight or overweight category have more complications such as hypertensive syndrome, cesarean sections, preterm birth and the same can be observed in the baby in the short or long term such as macrosomia, intrauterine growth restriction, need for hospitalization in intensive care units, and fetal distress.^(3)^ In cases where the woman is underweight, she is at higher risk of developing miscarriages, anemia, nutritional deficiencies, and the baby may be born with low weight, premature and with birth defects.^(4)^

Body composition is the ratio between the different body components and total body mass, expressed as percentages of fat and lean mass.^(4)^ Through its evaluation, we determine the components of the human body quantitatively, and it is useful to monitor the degree of development and growth, the state of the body components of different population groups, including pregnant women.^(4)^ Body fat percentage can be estimated by the sum of skinfold measurements.^(5)^

The nutritional and metabolic changes that occur during pregnancy aim to provide a favorable environment for the adequate development of the fetus.^(6)^ Pregnant women are susceptible to nutritional inadequacy due to the increased demand for macronutrient energy that is essential for the synthesis of new tissues and growth of existing tissues, in parallel there is the adjustment of protein metabolism, greater insulin resistance that occurs in this phase, since the pancreatic beta cells cannot compensate satisfactorily, glucose metabolism is altered and contributes to the onset of gestational diabetes mellitus (GDM).^(5,7,8)^ GDM increases the chances of macrosomia, metabolic disorders, growth imbalance, hyperbilirubinemia, cardiovascular defects, stillbirths, and perinatal death.^(9)^

In 1921, Matiegka^(10)^ created the first body assessment model based on the use of skinfolds, which aimed to qualify individuals to work in the industry, having expanded its applicability with studies that demonstrated the association with cancer, diabetes, cardiovascular diseases, and osteoporosis.

Skinfold thickness measurement is a method used to assess nutritional status, reflecting the distribution of subcutaneous fat in various locations.^(2–5)^ It aims to measure adiposity, considering that it measures two layers of the skin together with the fat of a specific point, considering that more than half of the body fat, about 50% to 70%, is subcutaneous and is in the subcutaneous deposits and has a relationship with the total weight of the body.^(11)^

In their findings, Boucher et al.^(12)^ and Kannieappan et al.^(13)^ agree that, in isolation, BMI has limitations because it does not distinguish the constituents of body composition, is not able to predict cases of false skinny skin, individuals with metabolically healthy obesity, nutritional risk situations as well as skinfold thickness. The skinfold allows an in-depth assessment of body composition, through which it is possible to assess the deposition of fat in different parts of the body and use of 2 to 5 points is usually sufficient to assess body distribution.^(14,15)^

In this context, the nutritional assessment and monitoring of nulliparous women can contribute significantly to the maintenance of an adequate gestational weight for both women and the baby, influencing gestational outcomes. In addition, the use of skinfolds as a risk indicator in pregnant women can facilitate the early identification of complications, allowing preventive interventions that promote a healthier pregnancy.

This study aims to evaluate the sum of skinfolds in nulliparous women and the risk of complications during pregnancy, emphasizing the importance of deepening studies on the dynamics/trajectory of skinfold thickness throughout pregnancy, considering the opportunities that the assessment of nutritional status through skinfolds can provide, especially in a scenario where the scientific literature lacks studies in this approach.

## Methods

We used data from two multicenter longitudinal prospective cohort studies conducted in Brazil, the Preterm SAMBA study (Preterm Screening and Metabolomics in Brazil and Auckland) and the MAES study (Maternal Actigraphy Exploratory Study). These cohorts followed nulliparous pregnant women at usual risk, with no previous history of severe disease, between 19 and 21 weeks of gestation until delivery in 5 and 4 maternity hospitals, respectively. The methodological information has been described in detail.^(16,17)^ Briefly, the Preterm SAMBA and MAES studies included women from July 2015 to July 2018 and from March 2018 to June 2020, respectively, in obstetric reference maternity hospitals in three regions of the country: Northeast, Southeast, and South.

Women with a previous history of three abortions, cervical abnormalities, major fetal anomaly, history of cervical conization with a scalpel, chronic corticosteroid use, and detected or self-reported preexisting disease, including hypertensive disease, previous diagnosis of diabetes mellitus, renal disease, systemic lupus erythematosus disease or antiphospholipid syndrome, sickle cell anemia, and positive serology for HIV (Human Immunodeficiency Virus) were excluded, and the same for women who were taking medications or supplements that could interfere with the outcome assessment, such as aspirin, calcium, fish oil, vitamin C, vitamin E, or heparin.^(17)^

Data from women who participated in the first visit of the study, women with data duly filled out in the SAMBA and MAES databases, were included in the study.

To assess nutritional status, all centers used an electronic scale and a calibrated anthropometer and measurements were taken throughout all visits.

In all centers, trained professionals from the health team performed the woman's anthropometric measurements (weight, height, skinfolds, and arm circumference) in the three follow-up visits of the study during pregnancy. Study visits were conducted in weeks 19–21 (1st study visit), 27–29 (2nd study visit), and 37–39 (3^rd^ study visit). All measurements were taken three times and recorded as rounded values according to standardized criteria defined by the Food and Nutrition Surveillance System of the Ministry of Health.^(17)^

Regarding the anthropometric assessment, the BMI calculated at the beginning of the study between 19 and 21 weeks of gestation was used according to Atalah et al. (1997).^(18)^ Women were classified according to their BMI in the first study visit into underweight (below 18.5kg/m²), adequate (between 18.5 – 24.9kg/m²), overweight (between 25 – 29.9kg/m²) and obese (>30kg/m²).

At the three study visits during pregnancy and at the postnatal visit, additional information was collected on maternal history, details of pregnancy complications, maternal biophysical data (weight, height, skinfolds), and adverse pregnancy outcomes.^(16)^

Trajectory for those who had a small for gestational age newborn, preeclampsia, preterm birth (which only 1st and 2nd visits were considered), no major complications, and GDM were evaluated.

The Brazilian Ethical Resolutions were complied with, especially Resolution No. 466, of December 12, 2012, of the National Health Council and the commitment to safeguard the safety and well-being of those involved. After including all ethical considerations, the Informed Consent term was presented and signed by the women included in the original study. In this term, participants who agreed to the use of their data for future studies without the need to sign another informed consent form were included. This article follows the STROBE (Strengthening the Reporting of Observational Studies in Epidemiology) checklist for reporting a cohort study.^(19)^

The characterization of skinfolds was based on the quartiles established at the 1st study visit, resulting in values in millimeters and the quartiles were considered <Q1 (1st quartile: values less than 25%) as lower body fat percentage, Q1 – Q3 (2nd quartile: values between 25% and 75% or the median) as average body fat percentage, and >Q3 (3rd quartile: values above 75% to 100%) as higher percentage of body fat.

To compare sociodemographic differences, the χ2 test was applied for categorical variables, and for numerical variables, the Mann-Whitney test and the Shapiro-Wilk normality test were applied. All results with a P<0.05 value was considered significant. To measure the strength of the association between skinfolds and risk for adverse outcomes, analyses of the odds ratio of the occurrence of the investigated outcome were performed. Statistical analyses, harmonization and data mining were performed using the software R version 4.2.2 (2022-10-31 ucrt) - "Innocent and Trusting".^(20)^ Graphs were created using R software and GraphPad Prism version 9.0 (GraphPad Software, La Jolla, CA, USA).

It was approved by the Research Ethics Committees of all participating centers 912.714 (coordinating center protocol - *Certificado de Apresentação para Apreciação Ética*: CAAE #20182318.8.0000.5404), in addition to the National Research Ethics Committee.

## Results

In total, 1,565 nulliparous women were enrolled in the Preterm SAMBA and MAES cohort studies. In this analysis, 38 were excluded due missing information of skinfolds in the first study visit. Finally, 1,527 nulliparous women were considered in the analysis (Figure 1).

**Figure 1:**
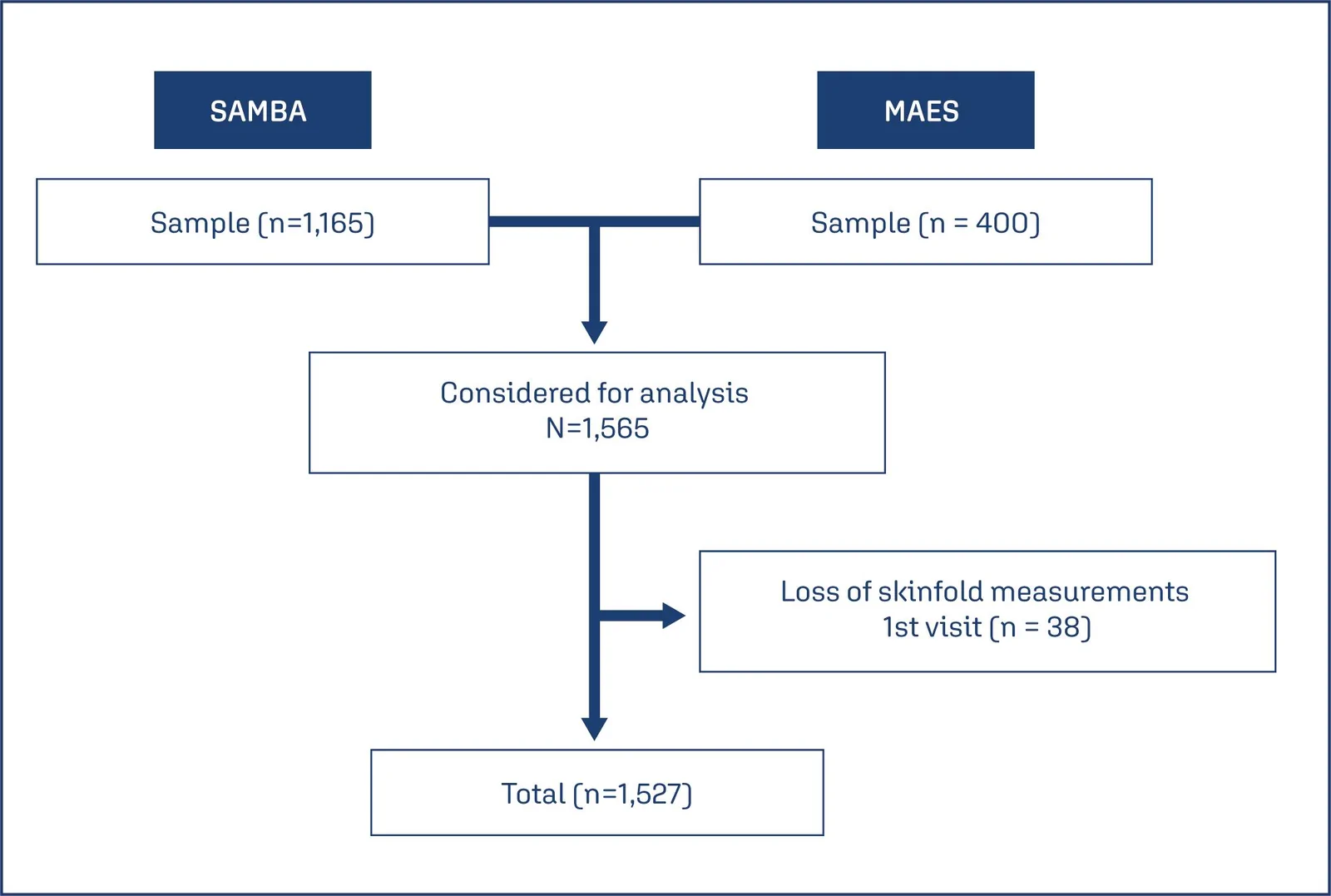
Flowchart of study population (Preterm SAMBA and MAES-I studies)

Data of skinfolds were available for 1,270 nulliparous in the second visit (16.83% loss) and 803 for the third visit (47.41% loss). The main reason for losses includes preterm birth 10.8% and loss of follow-up of which 6.8% on the first visit, 23.11% on the second visit and 36.86% on the third visit. Table 1 show the sociodemographic, anthropometric and pregnancy characteristics. Most of the study participants were non-white (61.0%), had between 20 and 35 years old (71.6%), had 9-12 years of schooling (58.0%), had a partner (73.0%) and had monthly income over R$ 12,000 (41.4%). In the first study visit, the subscapular, triceps and biceps skinfold mean (±standard deviation) were 21.1 (±8.6), 21.4 (±7.7), and 15.0 (±6.9), respectively. The mean of the sum of the skinfold at the first study visit was 78.0 (±28.6).

**Table 1:** Characteristics of the study population

Maternal characteristics			n(%) or Mean (±SD)
Age			
	<20 Years		367(24.0)
	20-35 Years		1,093(71.6)
	>35 Years		67(4.4)
Color/Ethnicity			
	White		596(39.0)
	Non-white		931(61.0)
Region			
	Northeast		717(47.0)
	South/Southeast		810(53.0)
Marital status			
	With partner		1,116(73.0)
	Without partner		411(27.0)
Schooling			
	≤8 Years		188(12.0)
	9-12 Years		888(58.0)
	>12 Years		451(30.0)
	Income (R$ per month)		
	Level I		128(8.4)
	Level II		290(19.0)
	Level III		477(31.2)
	Level IV		632(41.4)
Maternal BMI – V1			
	Adequate		598(39.0)
	Obese		255(17.0)
	Overweight		412(27.0)
	Underweight		262(17.0)
Skinfolds			
	Skinfold subscapular 1st visit		21.1(8.6)
	Skinfold subscapular 2nd visit^a^		22.5(8.3)
	Skinfold subscapular 3rd visit^b^		23.1(8.5)
	Skinfold triceps 1st visit		21.4(7.7)
	Skinfold triceps 2nd visit^a^		22.2(7.5)
	Skinfold triceps 3rd visit^b^		22.4(7.7)
	Skinfold biceps 1st visit		15.0(6.9)
	Skinfold biceps 2nd visit^a^		15.7(6.9)
	Skinfold biceps 3rd visit^b^		16.2(6.9)
	Sum of the skinfolds 1st visit		73.5(29.0)
	Sum of the skinfolds 2nd visit^a^		76.6(28.5)
	Sum of the skinfolds 3rd visit^b^		78.0(28.6)
Pregnancy outcome^c^			
	Gestational diabetes mellitus		134(8.8)
	Preeclampsia		109(7.2)
	Small for gestational age		155(10.0)
	Preterm birth		163(10.8)
		Spontaneous	101(6.7)
		Therapeutic	62(4.1)
Birth weight^d^			3,133.88 (±584.89)
Newborn length^e^			48.12 (±3.24)

Missing information for

a) 258;

b) 467;

c) 11;

d) 45;

e) 119.

Family income: Level I - Low (Up to R$500/month), Level II - Low Medium (R$ 501 - R$ 1.000/month), Level III - Medium High (R$ 12 thousand - R$ 24 thousand), Level IV - High (> R$ 24 thousand)

Table 2 describes the distribution of each skinfold for every study visit. In general, there was an increase of all skinfolds over the three study visits.

**Table 2:** Distribution of the sum of skinfolds according to the study visits

Skinfolds (mm)		Study visits		
		19-21weeks	27-29 weeks	37-39 weeks
Subscapular skinfolds	Mean (sd)	21.1 (8.6)	22.5 (8.3)	23.1 (8.5)
	5th centile	10.0	11.0	11.0
	10th centile	11.0	12.3	13.0
	25th centile	14.0	16.0	17.0
	50th centile	20.0	22.0	22.0
	75th centile	26.0	27.0	29.0
	90th centile	33.0	33.0	34.2
	95th centile	37.0	37.0	39.0
Triceps skinfolds	Mean (sd)	21.4 (7.7)	22.2 (7.5)	22.4 (7.7)
	5th centile	10.2	11.2	11.3
	10th centile	11.9	12.7	12.5
	25th centile	15.5	17.0	17.0
	50th centile	20.5	21.0	22.0
	75th centile	26.0	27.0	27.0
	90th centile	32.0	32.0	33.0
	95th centile	35.7	36.0	37.0
Biceps skinfolds	Mean (sd)	15.0 (6.9)	15.7 (6.9)	16.2 (6.9)
	5th centile	6.5	7.0	7.0
	10th centile	8.0	8.0	9.0
	25th centile	10.0	10.9	11.0
	50th centile	13.0	14.0	15.0
	75th centile	19.0	20.0	20.0
	90th centile	25.0	25.0	26.0
	95th centile	29.0	30.0	30.0
Sum of skinfolds	Mean (sd)	73.5 (29.0)	76.6 (28.5)	78.0 (28.6)
	5th centile	34.5	37.9	38.0
	10th centile	40.0	43.6	43.4
	25th centile	51.1	55.0	55.5
	50th centile	69.0	73.0	74.0
	75th centile	91.0	94.3	96.0
	90th centile	114.0	115.1	117.0
	95th centile	127.8	128.2	128.0

Sum of skinfolds: Sum of the subscapular, triceps, and biceps skinfold; all mean and median comparisons showed p-values = <0.05

The variation of skinfolds throughout pregnancy differed according to maternal characteristics. The higher the subgroup of age, the higher the initial sum of the skinfolds at the first study visit (Figure 2).

**Figure 2:**
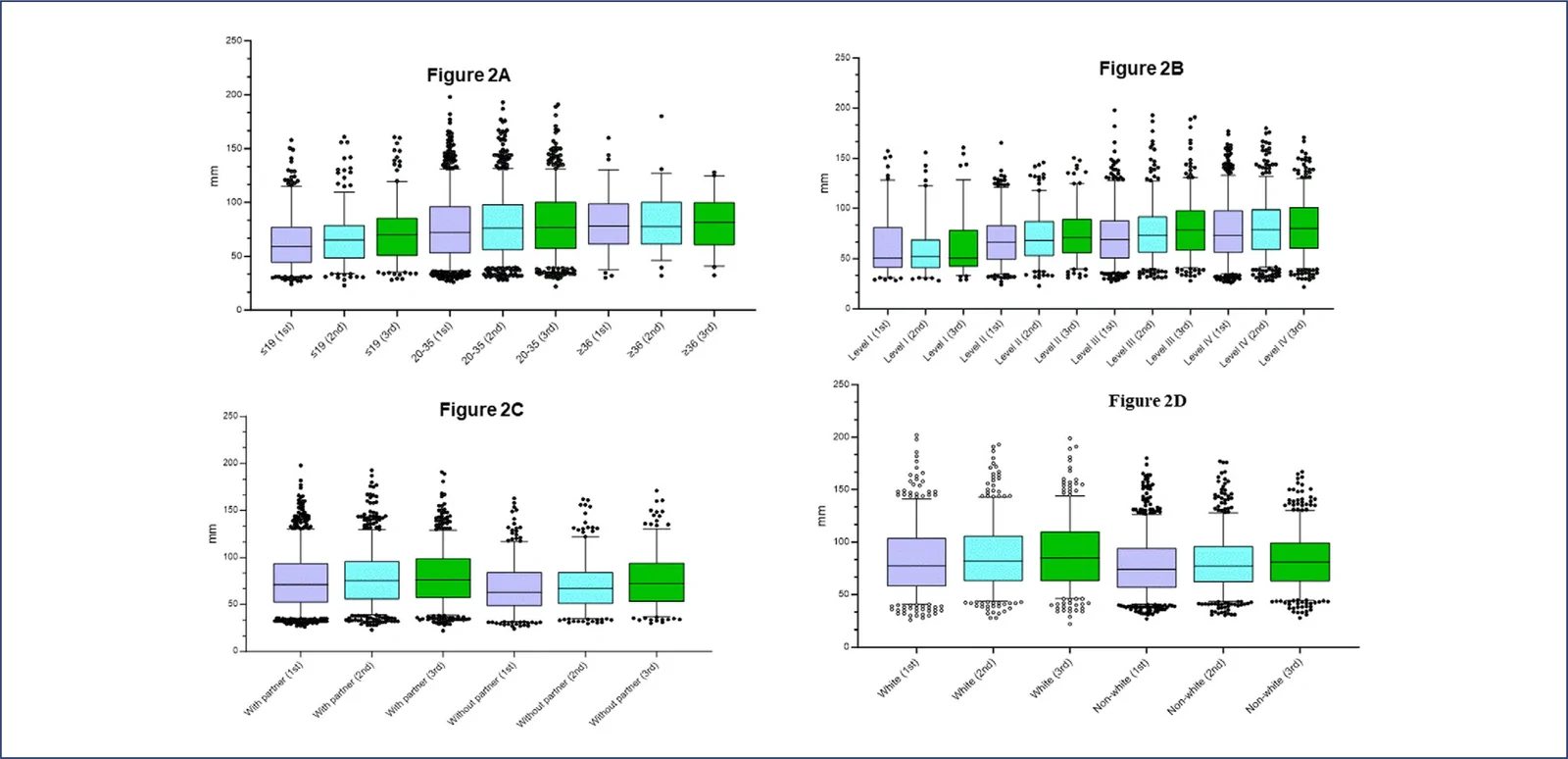
Sum of skinfolds by maternal characteristics in each study visit

Figure 2A. Maternal age in years (p-value = <0.0001 in all reviews). Figure 2B. Family income: Low (Up to R$500/month), Low-medium (R$ 501 - R$ 1.000/month), Medium-high (R$ 12 thousand - R$ 24 thousand), High (> R$ 24 thousand), (p-value = <0.0001 for all comparisons; The different categories for the same visit and the same category for different study visits were compared). Figure 2C. Marital status (p-value = <0.0001 at the first and second study visits and 0.093 at the third), Figure 2D. Skin color (p-value = 0.001 in the first assessment and <0.0001 in the next two)

All age subgroups (<19, 20-35 and >35y) showed an increase on the sum of the skinfolds over the study visits. Similarly, the increase of the sum of the skinfolds were demonstrated in all subgroups of maternal characteristics, except for level I of income, whose skinfolds had decreased between the second and third trimester (Figure 2). Detailed information on all skinfold's measurements by maternal and pregnancy characteristic are shown in the Supplementary Material Table 1S-5S.

In figure 3 we showed the distribution of skinfolds according to BMI categories and the study visits. The sum of skinfold at the initial study visit (19-21wks) was higher in the groups with a higher BMI (skinfold from obese > overweight > adequate > underweight; p<0.001). There was a significant increase of the skinfold for all BMI subgroups in the second and third study visits, although it was less relevant in the obese subgroup.

**Figure 3:**
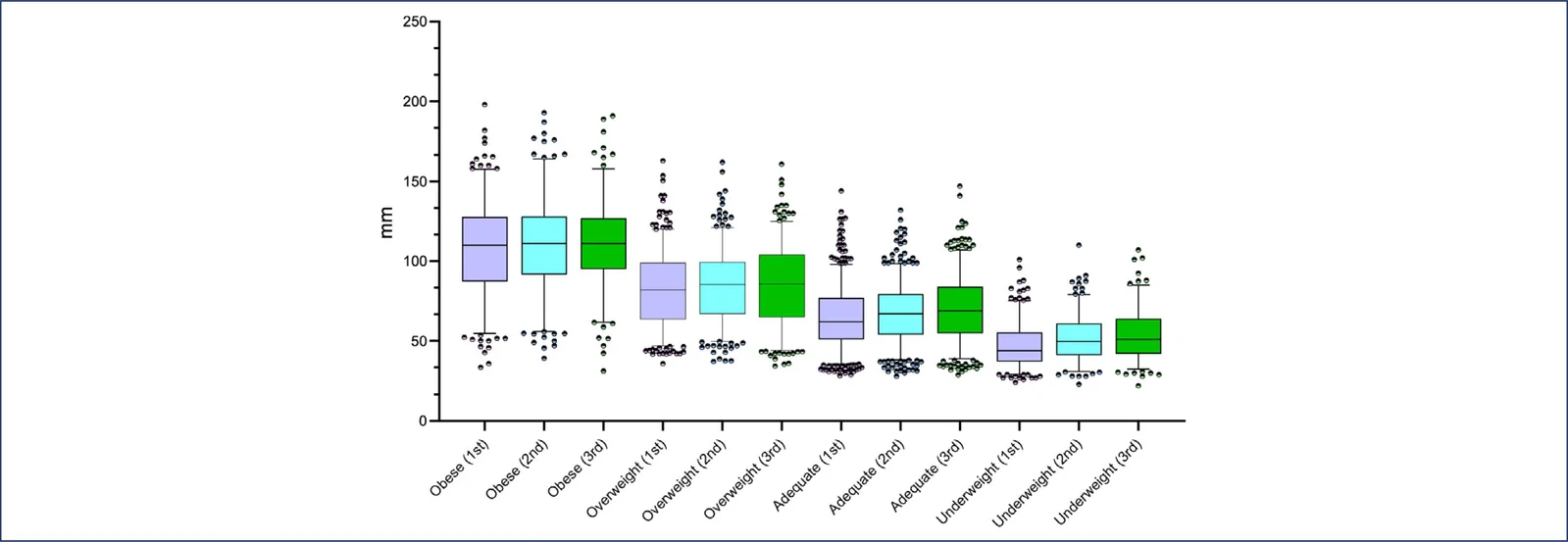
Distribution of skinfolds according to BMI categories in each study visit

The classification of nutritional status was made using the calculation of BMI at the first prenatal visit according to Atalah et al.^(18)^ Different categories were compared at each visit, and comparisons of the same category were compared for different study visits (p-value= <0.0001 in all assessments).

In figures 4, 5, 6 and 7, we demonstrated that the trajectory of the sum of skinfolds varied according to the pregnancy outcomes and the respective maternal BMI. Only women with no major complication showed an increase of the sum of the skinfold in the second study visit regardless the maternal BMI, while women with complications had different trajectories depending on the maternal BMI. Women with obesity, adequate BMI and overweight women who presented preeclampsia (PE) increased the sum of skinfolds between the first and the second visits, while underweight women with preeclampsia decreased. Detailed information on all skinfolds measurements by pregnancy outcomes subgroups are shown in the Supplementary Material, Tables 6S-9S.

**Figure 4:**
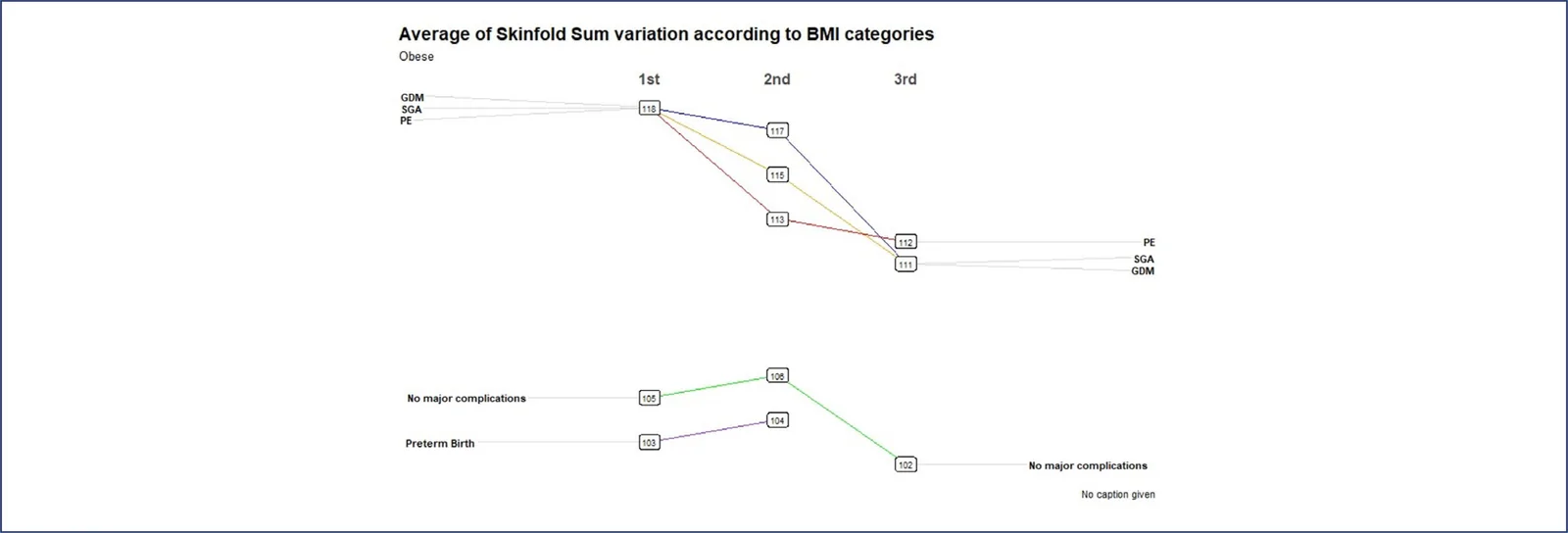
Sum of skinfolds in each study visit according to pregnancy outcome in obese women (1^st^ visit n= 255, 2^nd^ visit n= 24, 3^rd^ visit n= 247)

**Figure 5:**
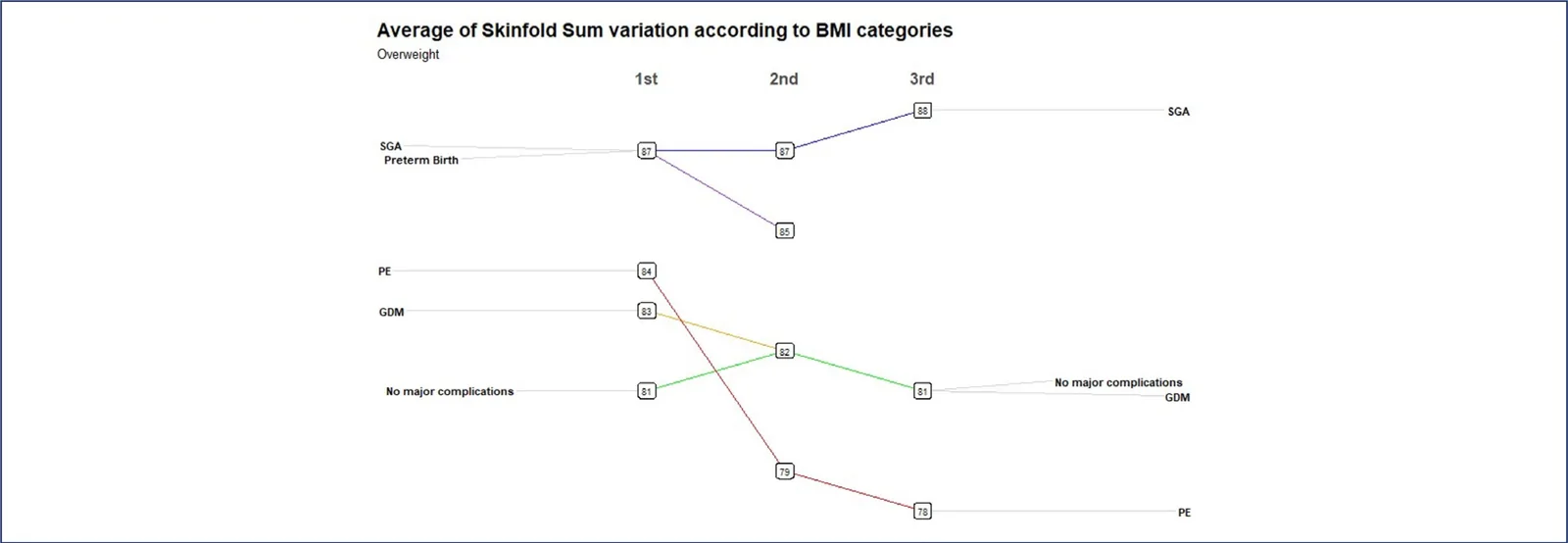
Sum of skinfolds in each study visit according to pregnancy outcome in overweight women (1^st^ visit n= 412, 2^nd^ visit n= 369, 3^rd^ visit n= 333)

**Figure 6:**
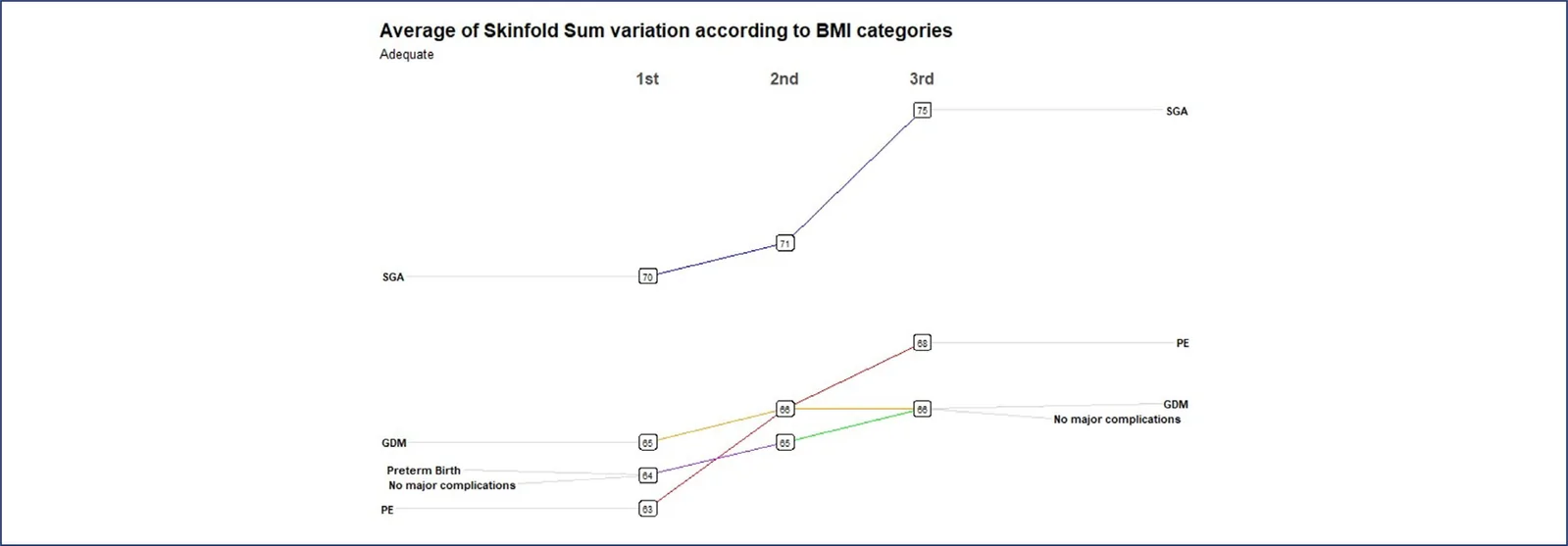
Sum of skinfolds in each study visit according to pregnancy outcome in women with adequate BMI (1^st^ visit n= 598, 2^nd^ visit n= 480, 3^rd^ visit n= 366)

**Figure 7:**
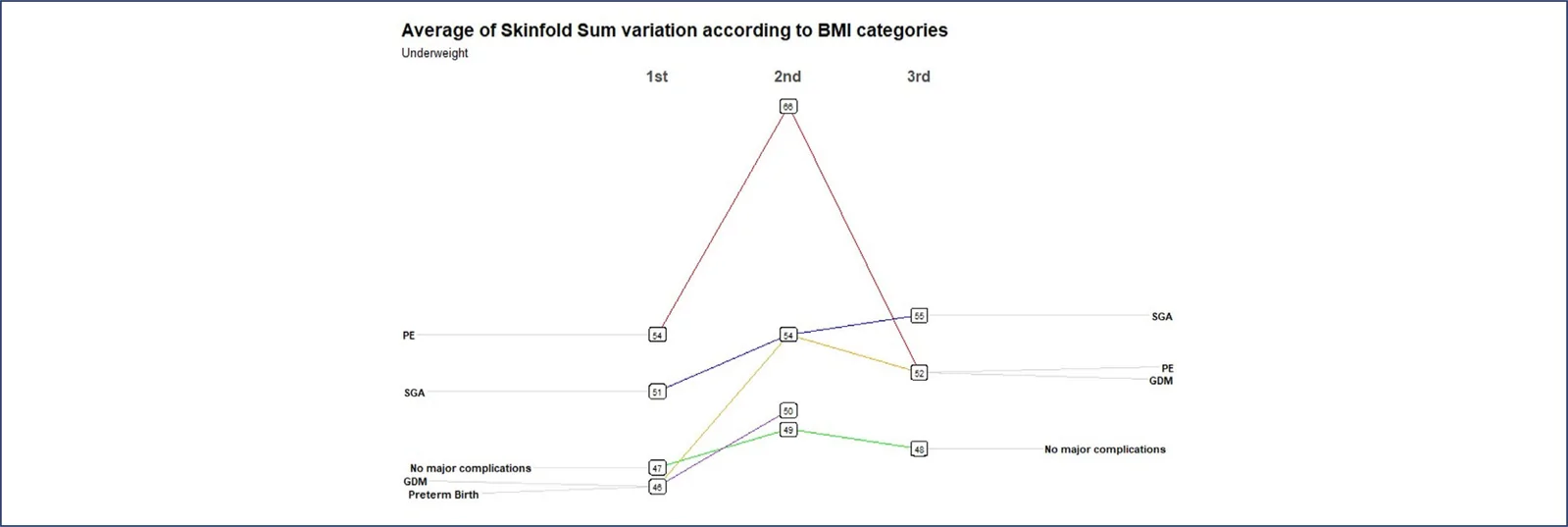
Sum of skinfolds in each study visit according to pregnancy outcome in underweight women (1^st^ visit n= 262, 2^nd^ visit n= 177, 3^rd^ visit n= 119)

## Discussion

Our results suggest that women who are underweight, adequate and overweight tend to gain more skinfold weight throughout pregnancy compared to women with obesity. The assessment of the pregnant woman's skinfolds during the study visits showed significant variability for women with gestational diabetes mellitus and small for gestational age. This demonstrates the importance of monitoring the nutritional status of nulliparous women by combining the methods of nutritional status assessment and skinfold. Currently, there are a lot of studies that explore the assessment of body fat throughout pregnancy and its relationship with pregnancy outcomes, in the routine assessment of the pregnant woman's body composition.^(13,21,22)^

In our study, most of the participants lived in the south/southeast of the country and did not have a partner, geographic location has been discussed in many studies as a factor strongly related to the nutritional status of individuals, but we considered the improvement of actions aimed at food and nutrition education during the gestational period. Taking into account this aspect, since when we look at the population residing in the South region, we observe a population with easier access to public services, employment, education, physical activity and access to healthy food.

The pattern of changes in arm circumference, subscapular skinfolds, biceps and triceps during the course of pregnancy and their maternal and neonatal outcomes in a prospective cohort of 1066 pregnant women in 7 urban regions in Argentina, the mean total increases in skinfolds were 4.5, 3.6 and 2.6 mm from 16 to 36 weeks of gestation, a finding that is similar to the variations found in our study.^(21)^ In addition, obese women had smaller increases in mean skinfold values compared to underweight, adequate, and overweight women.^(21)^ This may be justified by the fact that these women constitute a risk group that justifies this reduced gain compared to women who are within the standard considered adequate, or even because possibly they are already aware of their nutritional status and take more care with their diet. We also emphasize that our values were similar to those of these authors.

When we look at nulliparous women taking into account age, we observe that women under 19 years of age gain more skinfold weight compared to women over 20 to 35 years of age and those over 35 years of age, a fact that can be justified by the fact that older women have more associated comorbidities such as hypertension, being more prone to stress, the use of medications, a more sedentary lifestyle and not practicing physical activity or even by the fact that the younger ones are at a stage in which the highest metabolic rate, the stage in which they are, provides greater growth and development.^(5)^ Which, in a way, ends up corroborating that they have a greater fold compared to women from other groups.

In a prospective cohort of 1820 pregnant women, fat mass loss between the third trimester and postpartum period was similar among women in the underweight, adequate and overweight categories, while obese women lost significantly less fat mass than those in the other BMI groups (p=0.003).^(22)^ This finding supports the results we obtained in our study, in which underweight women moved from low fat percentage gain to medium fat percentage and obese women remained in the range of excess body fat throughout all study visits.

Weight gain can be considered crucial in the monitoring of pregnant women, especially when looking at risk factors such as nulliparity. The assessment of pre-gestational nutritional status has been shown to be effective in reducing the rate of weight gain in obese pregnant women, which may support the finding that women with obesity did not present greater increases in skinfold values over the two gestational trimesters.^(23)^ This finding is supported by Widen and Gallagher^(22)^ in which, in order to accurately predict changes in the body composition of pregnant women, it is necessary that the measurements occur close to conception.

In addition, a strong correlation was observed between skinfold thickness and the development of gestational diabetes mellitus, which had an impact on maternal and infant outcomes, and considerable cases of macrosomia were found in more than half (54%) of the newborns in the 5 centers where the study was conducted.^(24)^ Our findings regarding GDM call attention due to the negative impacts that this outcome can bring to the pregnant woman and the fetus, serious alterations such as congenital malformations, alterations that will interfere with fetal development.

For women of childbearing age, it was found that pre-gestational nutritional status is a factor associated with excessive gestational weight gain. The sample studied consisted of women with a high prevalence of pre-gestational overweight and a considerable portion of them had excessive weight gain during pregnancy.^(25)^ The need to improve the monitoring of pregnant women to provide them with a healthier pregnancy, because knowing their nutritional status and giving the proper follow-up, there is a great opportunity to have positive outcomes for both.

Studies have demonstrated a correlation between body composition at the beginning of pregnancy and the development of gestational diabetes mellitus and found that weight gain in early pregnancy is closely related to gestational diabetes mellitus, and that excessive weight gain in early pregnancy increases the risk of GDM by 4.08 times and is an independent risk factor for GDM.^(26,27)^ It is also noteworthy that a pre-gestational BMI of 24kg/m² indicated an increase in weight in pregnant women in the first trimester, which increased the risk of GDM by 5.19 times.^(25,28)^ In our findings, we found a strong association between BMI and GDM.

Health and nutrition indicators reveal the ongoing income and race disparities within the country: Black and low-income women exhibit higher rates of chronic diseases compared to their white counterparts of similar age and higher income levels. This finding supports the fact that non-white women gained more skinfold weight compared to white women, which may be related to years of schooling, lower income and, to a certain extent, health care and nutrition.^(29)^

There was no difference in weight gain between women who started pregnancy with low weight, adequate or overweight, only women with BMI in the obesity range had significantly lower weight gain.^(30,31)^

One of the limitations found was the existence of few articles addressing the variation of skinfold thickness in nulliparous pregnant women over two gestational trimesters, implying the use of very old literature and especially the need for more studies related to the topic under discussion, to strengthen communication and create new policies that favor women.

We can highlight the strengths of our study such as the diversity we were able to obtain with our population, since we work with women from 3 regions of Brazil, South, Southeast and Northeast, the approach enhances the evaluation of pregnant women's body composition throughout their pregnancy by incorporating skinfold measurements into existing protocols.

## Conclusion

In conclusion, our study suggest that the trajectory of skinfolds differ according to maternal characteristics, especially maternal BMI and monitoring their change over pregnancy may be a resourceful tool in identifying women who may develop pregnancy complications and was associated with gestational diabetes mellitus and intrauterine growth restriction. With these findings we recommend that more studies be developed in order to ensure a complete and reliable evaluation and surveillance of pregnant women. An increase in the sum of skinfold thickness was observed between 20-27 and 27-37 weeks in nulliparous pregnant.

## Data Availability

The research data are described in the article presented.
