# Supplementary material for: Skinfolds in nulliparous women and their association with pregnancy complications: a multicenter cohort study in Brazil
Source: Rev Bras Ginecol Obstet. 2026 Oct 30;48:e-rbgo31. doi: 10.61622/rbgo/2026rbgo31 (PMC13754077; doi:10.61622/rbgo/2026rbgo31)
Supplement: Supplementary file 1 [file 1806-9339-rbgo-48-e-rbgo31-Suppl01.pdf]

Supplementary material

Table 1S. Sum of skinfolds according to maternal characteristics – MATERNAL AGE

Characteristic	Maternal age			p-value#
	20-35years* n=1,093	≤19years* n=367	≥36years* n=67	
Skinfold subscapular 1st visit	22.1(8.8)	18.0(7.3)	22.5(10.0)	<0.001
Skinfold subscapular 2nd visit	23.3(8.4)	19.8(7.3)	23.3(9.1)	<0.001
Unknown	174	73	11	
Skinfold subscapular 3rd visit	23.9(8.8)	21.1(7.5)	22.2(7.7)	<0.001
Unknown	327	122	21	
Skinfold triceps 1st visit	22.5(7.9)	18.2(6.7)	22.1(6.4)	<0.001
Skinfold triceps 2nd visit	23.1(7.5)	19.3(6.8)	23.0(6.8)	<0.001
Unknown	173	73	11	
Skinfold triceps 3rd visit	23.3(7.9)	20.1(7.1)	22.0(5.9)	<0.001
Unknown	325	121	21	
Skinfold biceps 1st visit	15.6(7.1)	13.0(6.0)	16.9(7.3)	<0.001
Skinfold biceps 2nd visit	16.2(7.0)	13.9(6.0)	17.7(8.5)	<0.001
Unknown	173	73	11	
Skinfold biceps 3rd visit	16.6(7.1)	14.9(6.5)	16.7(6.8)	<0.001
Unknown	325	121	21	
Sum of the 3 skinfolds 1st visit	76.6(29.7)	63.4(24.6)	79.5(26.6)	<0.001
Sum of the 3 skinfolds 2nd visit	79.5(29.1)	67.0(24.4)	82.2(27.5)	<0.001
Unknown	173	73	11	
Sum of the 3 skinfolds 3rd visit	80.3(29.3)	71.1(26.1)	78.8(23.6)	<0.001
Unknown	325	121	21	
Schooling				<0.001
>12 Years	401/1,093(37)	6/367(1.6)	44/67(66)	
≤8 Years	58/1,093(5.0)	126/367(34)	4/67(6.0)	
9-12 Years	634/1,093(58)	235/367(64)	19/67(28)	
Income				<0.001
High	520/1,093(48)	70/367(19)	42/67(63)	
Low	82/1,093(7.5)	39/367(11)	7/67(10)	
High average	326/1,093(30)	136/367(37)	15/67(22)	
Low average	165/1,093(15)	122/367(33)	3/67(4.5)	
Civil				<0.001
With partner	873/1,093(80)	186/367(51)	57/67(85)	
No partner	220/1,093(20)	181/367(49)	10/67(15)	
Region				0.040
Northeast	508/1,093(46)	186/367(51)	23/67(34)	
South/Southeast	585/1,093(54)	181/367(49)	44/67(66)	
GDM				<0.001
No	981/1,086(90)	347/364(95)	54/66(82)	
Yes	105/1,086(9.7)	17/364(4.7)	12/66(18)	
Unknown	7	3	1	
PE				0.13
No	1,011/1,086(93)	339/364(93)	57/66(86)	
Yes	75/1,086(6.9)	25/364(6.9)	9/66(14)	
Unknown	7	3	1	
SGA				0.7
No	971/1,086(89)	329/364(90)	61/66(92)	
Yes	115/1,086(11)	35/364(9.6)	5/66(7.6)	
Unknown	7	3	1	
Preterm birth				0.2
No	964/1,086(89)	333/364(91)	56/66(85)	
Yes	122/1,086(11)	31/364(8.5)	10/66(15)	
Unknown	7	3	1	
Spontaneous				0.7
No	1,003/1,075(93)	336/359(94)	60/66(91)	
Yes	72/1,075(6.7)	23/359(6.4)	6/66(9.1)	
Unknown	18	8	1	
Therapeutic				0.062
No	1,017/1,067(95)	349/357(98)	61/65(94)	
Yes	50/1,067(4.7)	8/357(2.2)	4/65(6.2)	
Unknown	26	10	2	

Continue...

Continuation.

Characteristic	Maternal age 20-35years* n=1,093	≤19years* n=367	≥36years* n=67	p-value#
Parturition				
Elective cesarean section	269/1,086(25)	52/364(14)	32/66(48)	
Non-elective cesarean section	271/1,086(25)	65/364(18)	15/66(23)	
Vaginal delivery	519/1,086(48)	240/364(66)	16/66(24)	
Surgical delivery	27/1,086(2.5)	7/364(1.9)	3/66(4.5)	
Unknown	7	3	1	
Apgar7				0.4
No	1,031/1,046(99)	337/345(98)	63/64(98)	
Yes	15/1,046(1.4)	8/345(2.3)	1/64(1.6)	
Unknown	47	22	3	
Weight newborn	3,131.7(579.9)	3,139.0(588.2)	3,142.0(653.4)	0.6
Unknown	29	14	2	
Length newborn	48.1(3.1)	48.1(3.3)	47.9(4.6)	0.4
Unknown	82	33	4	

*Mean (SD); n / n(%)

#Kruskal-Wallis rank sum test; Pearson's Chi-squared test; Fisher's exact test

Table 2S. Sum of skinfolds according to maternal characteristics - SCHOOLING

Characteristic	Schooling ≤8 years* n = 188	9-12 years* n = 888	>12 years* n = 451	p-value#
Skinfold subscapular 1st visit	18.8(7.6)	21.5(8.6)	21.5(9.2)	<0.001
Skinfold subscapular 2nd visit	21.1(8.0)	22.8(8.1)	22.3(8.8)	0.061
Unknown	56	140	62	
Skinfold subscapular 3rd visit	21.4(7.9)	23.6(8.3)	22.8(9.0)	0.019
Unknown	87	257	126	
Skinfold triceps 1st visit	18.8(7.2)	21.3(7.6)	23.0(7.9)	<0.001
Skinfold triceps 2nd visit	19.8(7.3)	22.0(7.4)	23.4(7.5)	<0.001
Unknown	56	139	62	
Skinfold triceps 3rd visit	20.2(7.9)	22.5(7.5)	23.3(8.0)	0.002
Unknown	86	256	125	
Skinfold biceps 1st visit	13.8(7.0)	15.0(6.8)	15.6(7.1)	<0.001
Skinfold biceps 2nd visit	14.5(6.9)	15.8(6.9)	16.0(7.1)	0.016
Unknown	56	139	62	
Skinfold biceps 3rd visit	15.2(7.3)	16.4(6.9)	16.3(7.0)	0.10
Unknown	86	256	125	
Sum of the 3 skinfolds 1st visit	67.1(26.6)	73.7(29.0)	76.0(29.5)	0.001
Sum of the 3 skinfolds 2nd visit	71.0(26.5)	76.9(28.5)	78.2(29.0)	0.054
Unknown	56	139	62	
Sum of the 3 skinfolds 3rd visit	72.5(28.2)	78.9(28.8)	78.2(28.3)	0.11
Unknown	86	256	125	
Age				<0.001
≤19 Years	126/188(67)	235/888(26)	6/451(1.3)	
≥36 Years	4/188(2.1)	19/888(2.1)	44/451(9.8)	
20-35 Years	58/188(31)	634/888(71)	401/451(89)	
Income				<0.001
High	25/188(13)	295/888(33)	312/451(69)	
Low	26/188(14)	86/888(9.7)	16/451(3.5)	
High average	59/188(31)	325/888(37)	93/451(21)	
Low average	78/188(41)	182/888(20)	30/451(6.7)	
Civil				<0.001
With partner	112/188(60)	631/888(71)	373/451(83)	
No partner	76/188(40)	257/888(29)	78/451(17)	
Region				<0.001
Northeast	118/188(63)	409/888(46)	190/451(42)	
South/Southeast	70/188(37)	479/888(54)	261/451(58)	
GDM				0.10
No	175/185(95)	805/881(91)	402/450(89)	
Yes	10/185(5.4)	76/881(8.6)	48/450(11)	
Unknown	3	7	1	

Continue...

Continuation.

Characteristic	Schooling ≤8 years* n = 188	9-12 years* n = 888	>12 years* n = 451	p-value#
PE				0.7
No	169/185(91)	818/881(93)	420/450(93)	
Yes	16/185(8.6)	63/881(7.2)	30/450(6.7)	
Unknown	3	7	1	
SGA				0.4
No	783/881(89)	408/450(91)	170/185(92)	
Yes	98/881(11)	42/450(9.3)	15/185(8.1)	
Unknown	7	1	3	
Preterm Birth				0.7
No	168/185(91)	782/881(89)	403/450(90)	
Yes	17/185(9.2)	99/881(11)	47/450(10)	
Unknown	3	7	1	
Spontaneous				>0.9
No	173/185(94)	810/870(93)	416/445(93)	
Yes	12/185(6.5)	60/870(6.9)	29/445(6.5)	
Unknown	3	18	6	
Therapeutic				0.5
No	178/183(97)	825/864(95)	424/442(96)	
Yes	5/183(2.7)	39/864(4.5)	18/442(4.1)	
Unknown	5	24	9	
Parturition				
Elective cesarean section	28/185(15)	186/881(21)	139/450(31)	
Non-elective cesarean section	38/185(21)	206/881(23)	107/450(24)	
Vaginal delivery	117/185(63)	467/881(53)	191/450(42)	
Surgical delivery	2/185(1.1)	22/881(2.5)	13/450(2.9)	
Unknown	3	7	1	
Apgar 7				0.4
No	167/170(98)	836/853(98)	428/432(99)	
Yes	3/170(1.8)	17/853(2.0)	4/432(0.9)	
Unknown	18	35	19	
Weight newborn	3,171.1(642.3)	3,116.2(586.9)	3,153.0(555.8)	0.4
Unknown	7	30	8	
Length newborn	48.5(3.1)	48.0(3.3)	48.1(3.2)	0.3
Unknown	24	69	26	

*Mean (SD); n / n(%)
#Kruskal-Wallis rank sum test; Pearson's Chi-squared test; Fisher's exact test

Table 3S. Sum of skinfolds according to maternal characteristics – MARITAL STATUS

Characteristic	Marital status Withpartner* n=1,116	Nopartner* n=411	p-value#
Skinfold Subscapular 1st visit	21.8(8.9)	19.4(8.0)	<0.001
Skinfold Subscapular 2nd visit	23.1(8.5)	20.8(7.8)	<0.001
Unknown	176	82	
Skinfold Subscapular 3rd visit	23.5(8.6)	22.2(8.3)	0.020
Unknown	330	140	
Skinfold Triceps 1st visit	22.1(7.9)	19.8(7.2)	<0.001
Skinfold Triceps 2nd visit	22.6(7.6)	21.0(7.1)	<0.001
Unknown	175	82	
Skinfold Triceps 3rd visit	22.7(7.8)	21.8(7.5)	0.059
Unknown	328	139	
Skinfold Biceps 1st visit	15.3(7.0)	14.3(6.6)	0.009
Skinfold Biceps 2nd visit	16.0(7.1)	14.8(6.5)	0.004
Unknown	175	82	
Skinfold Biceps 3rd visit	16.4(7.1)	15.8(6.7)	0.3
Unknown	328	139	
Sum of the 3 Skinfolds 1st visit	75.5(29.6)	68.1(26.6)	<0.001
Sum of the 3 Skinfolds 2nd visit	78.5(29.0)	71.4(26.5)	<0.001
Unknown	175	82	
Sum of the 3 Skinfolds 3rd visit	79.0(28.8)	75.5(27.8)	0.076
Unknown	328	139	

Continue...

Continuation.

Characteristic	Marital status		p-value#
	Withpartner* n=1,116	Nopartner* n=411	
Age			<0.001
≤19 Years	186/1,116(17)	181/411(44)	
≥36 Years	57/1,116(5.1)	10/411(2.4)	
20-35 Years	873/1,116(78)	220/411(54)	
Schooling			<0.001
>12 Years	373/1,116(33)	78/411(19)	
≤8 Years	112/1,116(10)	76/411(18)	
9-12 Years	631/1,116(57)	257/411(63)	
Income			<0.001
High	491/1,116(44)	141/411(34)	
Low	98/1,116(8.8)	30/411(7.3)	
High Average	340/1,116(30)	137/411(33)	
Low Average	187/1,116(17)	103/411(25)	
Civil			0.049
With partner	507/1,116(45)	210/411(51)	
No partner	609/1,116(55)	201/411(49)	
Region			0.4
Northeast	1,007/1,109(91)	375/407(92)	
South/Southeast	102/1,109(9.2)	32/407(7.9)	
GDM			0.4
No	1,007/1,109(91)	375/407(92)	
Yes	102/1,109(9.2)	32/407(7.9)	
Unknown	7	4	
PE			0.9
No	1,030/1,109(93)	377/407(93)	
Yes	79/1,109(7.1)	30/407(7.4)	
Unknown	7	4	
SGA			0.8
No	997/1,109(90)	364/407(89)	
Yes	112/1,109(10)	43/407(11)	
Unknown	7	4	
Preterm Birth			0.5
No	993/1,109(90)	360/407(88)	
Yes	116/1,109(10)	47/407(12)	
Unknown	7	4	
Spontaneous			0.4
No	1,028/1,098(94)	371/402(92)	
Yes	70/1,098(6.4)	31/402(7.7)	
Unknown	18	9	
Therapeutic			0.8
No	1,041/1,087(96)	386/402(96)	
Yes	46/1,087(4.2)	16/402(4.0)	
Unknown	29	9	
Parturition			0.007
Elective cesarean section	276/1,109(25)	77/407(19)	
Non-elective cesarean section	265/1,109(24)	86/407(21)	
Vaginal Delivery	538/1,109(49)	237/407(58)	
Surgical Delivery	30/1,109(2.7)	7/407(1.7)	
Unknown	7	4	
Apgar 7			0.8
No	1,054/1,071(98)	377/384(98)	
Yes	17/1,071(1.6)	7/384(1.8)	
Unknown	45	27	
Weight_Newborn	3,144.9(580.0)	3,103.6(598.0)	0.4
Unknown	29	16	
Length_Newborn	48.2(3.1)	47.9(3.5)	0.14
Unknown	79	40	

*Mean (SD); n / n(%)
#Wilcoxon rank sum test; Pearson's Chi-squared test

Table 4S. Sum of skinfolds according to maternal characteristics – ANNUAL FAMILY INCOME

Characteristic	Annual Family Income				p-value#
	Up to R$ 6k* n = 128	R$ 6k - R$ 12k* n = 290	R$ 12k - R$ 24k* n = 477	> R$ 24k* n = 632	
Skinfold Subscapular 1st visit	19.5(7.6)	19.7(7.6)	20.8(8.2)	22.5(9.5)	<0.001
Skinfold Subscapular 2nd visit	19.8(6.9)	21.3(7.5)	22.2(7.8)	23.6(9.1)	<0.001
Unknown	28	72	83	75	
Skinfold Subscapular 3rd visit	20.6(7.4)	21.8(7.6)	23.5(8.5)	24.0(9.0)	<0.001
Unknown	32	108	163	167	
Skinfold Triceps 1st visit	18.8(8.3)	19.8(7.5)	21.1(7.6)	23.1(7.6)	<0.001
Skinfold Triceps 2nd visit	18.6(7.4)	20.4(7.3)	21.7(7.2)	23.9(7.4)	<0.001
Unknown	28	72	83	74	
Skinfold Triceps 3rd visit	19.4(8.1)	20.9(7.4)	22.8(7.3)	23.5(7.8)	<0.001
Unknown	31	108	163	165	
Skinfold Biceps 1st visit	14.3(7.4)	14.2(6.4)	15.0(6.9)	15.6(7.0)	0.002
Skinfold Biceps 2nd visit	14.6(7.4)	14.6(6.4)	15.9(6.9)	16.3(7.0)	<0.001
Unknown	28	72	83	74	
Skinfold Biceps 3rd visit	15.1(8.1)	15.3(6.1)	17.0(7.3)	16.3(6.7)	<0.001
Unknown	31	108	163	165	
Sum of the 3 Skinfolds 1st visit	62.1(30.8)	69.2(25.8)	73.0(28.0)	78.2(29.8)	<0.001
Sum of the 3 Skinfolds 2nd visit	60.1(28.0)	71.5(25.0)	76.5(27.7)	81.8(29.0)	<0.001
Unknown	28	72	83	74	
Sum of the 3 Skinfolds 3rd visit	63.3(31.2)	73.3(24.9)	80.3(28.9)	81.6(28.1)	<0.001
Unknown	31	108	163	165	
Age					<0.001
≤19 Years	39/128(30)	122/290(42)	136/477(29)	70/632(11)	
≥36 Years	7/128(5.5)	3/290(1.0)	15/477(3.1)	42/632(6.6)	
20-35 Years	82/128(64)	165/290(57)	326/477(68)	520/632(82)	
Schooling					<0.001
>12 Years	16/128(13)	30/290(10)	93/477(19)	312/632(49)	
≤8 Years	26/128(20)	78/290(27)	59/477(12)	25/632(4.0)	
9-12 Years	86/128(67)	182/290(63)	325/477(68)	295/632(47)	
Civil					<0.001
With partner	98/128(77)	187/290(64)	340/477(71)	491/632(78)	
No partner	30/128(23)	103/290(36)	137/477(29)	141/632(22)	
Region					<0.001
Northeast	47/128(37)	196/290(68)	241/477(51)	233/632(37)	
South/Southeast	81/128(63)	94/290(32)	236/477(49)	399/632(63)	
GDM					0.016
No	108/127(85)	272/290(94)	436/471(93)	566/628(90)	
Yes	19/127(15)	18/290(6.2)	35/471(7.4)	62/628(9.9)	
Unknown	1	0	6	4	
PE					0.9
No	117/127(92)	271/290(93)	434/471(92)	585/628(93)	
Yes	10/127(7.9)	19/290(6.6)	37/471(7.9)	43/628(6.8)	
Unknown	1	0	6	4	
SGA					0.036
No	115/127(91)	266/290(92)	407/471(86)	573/628(91)	
Yes	12/127(9.4)	24/290(8.3)	64/471(14)	55/628(8.8)	
Unknown	1	0	6	4	
Preterm Birth					0.7
No	115/127(91)	263/290(91)	415/471(88)	560/628(89)	
Yes	12/127(9.4)	27/290(9.3)	56/471(12)	68/628(11)	
Unknown	1	0	6	4	
Spontaneous					>0.9
No	117/124(94)	270/289(93)	436/466(94)	576/621(93)	
Yes	7/124(5.6)	19/289(6.6)	30/466(6.4)	45/621(7.2)	
Unknown	4	1	11	11	
Therapeutic					0.3
No	116/121(96)	278/286(97)	439/465(94)	594/617(96)	
Yes	5/121(4.1)	8/286(2.8)	26/465(5.6)	23/617(3.7)	
Unknown	7	4	12	15	

Continue...

Continuation.

Characteristic	Annual Family Income				p-value#
	Up to R$ 6k* n = 128	R$ 6k - R$ 12k* n = 290	R$ 12k - R$ 24k* n = 477	> R$ 24k* n = 632	
Parturition					
Elective cesarean section	28/127(22)	52/290(18)	100/471(21)	173/628(28)	
Non-elective cesarean section	33/127(26)	61/290(21)	112/471(24)	145/628(23)	
Vaginal Delivery	65/127(51)	176/290(61)	252/471(54)	282/628(45)	
Surgical Delivery	1/127(0.8)	1/290(0.3)	7/471(1.5)	28/628(4.5)	
Unknown	1	0	6	4	
Apgar 7					0.5
No	125/125(100)	268/272(99)	434/443(98)	604/615(98)	
Yes	0/125(0)	4/272(1.5)	9/443(2.0)	11/615(1.8)	
Unknown	3	18	34	17	
Weight_Newborn	3,150.1(666.3)	3,174.3(555.7)	3,090.5(618.7)	3,144.6(553.8)	0.5
Unknown	8	6	16	15	
Length_Newborn	48.1(3.4)	48.3(3.0)	48.0(3.7)	48.2(2.9)	>0.9
Unknown	17	19	40	43	

*Mean (SD); n / n(%)
#Kruskal-Wallis rank sum test; Pearson's Chi-squared test; Fisher's exact test

Table 5S. Sum of skinfolds according to maternal characteristics – BMI

Characteristic	BMI				p-value#
	Adequate* n = 598	Obese* n = 255	Overweight* n = 412	Underweight* n = 262	
Skinfold Subscapular 1st visit	18.4(5.7)	31.6(9.1)	24.0(6.3)	12.9(4.3)	<0.001
Skinfold Subscapular 2nd visit	19.8(5.7)	32.4(8.2)	25.3(6.1)	14.6(4.4)	<0.001
Unknown	97	38	76	47	
Skinfold Subscapular 3rd visit	20.6(6.1)	33.2(8.6)	25.7(6.9)	15.7(4.7)	<0.001
Unknown	164	88	126	92	
Skinfold Triceps 1st visit	19.1(5.4)	30.0(7.8)	24.4(6.1)	14.0(4.0)	<0.001
Skinfold Triceps 2nd visit	19.9(5.3)	30.6(7.5)	24.6(5.7)	15.3(4.5)	<0.001
Unknown	96	38	76	47	
Skinfold Triceps 3rd visit	20.4(5.9)	30.9(7.8)	24.7(6.4)	15.9(4.7)	<0.001
Unknown	164	86	126	91	
Skinfold Biceps 1st visit	12.7(4.8)	23.2(7.0)	16.8(5.9)	9.6(3.6)	<0.001
Skinfold Biceps 2nd visit	13.3(4.6)	23.8(7.4)	17.4(5.9)	10.6(4.0)	<0.001
Unknown	96	38	76	47	
Skinfold Biceps 3rd visit	14.1(5.2)	24.1(7.4)	17.7(6.0)	11.3(4.2)	<0.001
Unknown	164	86	126	91	
Sum of the 3 Skinfolds 1st visit	64.3(19.0)	108.3(29.2)	82.2(23.4)	47.2(14.2)	<0.001
Sum of the 3 Skinfolds 2nd visit	67.2(18.6)	110.7(29.2)	84.6(22.9)	52.0(15.0)	<0.001
Unknown	96	38	76	47	
Sum of the 3 Skinfolds 3rd visit	70.0(20.8)	111.6(27.9)	84.7(24.8)	54.5(16.4)	<0.001
Unknown	164	86	126	91	
Age					<0.001
≤19 Years	149/598(25)	29/255(11)	68/412(17)	121/262(46)	
≥36 Years	27/598(4.5)	12/255(4.7)	24/412(5.8)	4/262(1.5)	
20-35 Years	422/598(71)	214/255(84)	320/412(78)	137/262(52)	
Color/ethnicity					0.088
Non-white	356/598(60)	146/255(57)	272/412(66)	157/262(60)	
White	242/598(40)	109/255(43)	140/412(34)	105/262(40)	
Schooling					<0.001
>12 Years	196/598(33)	78/255(31)	125/412(30)	52/262(20)	
≤8 Years	75/598(13)	26/255(10)	39/412(9.5)	48/262(18)	
9-12 Years	327/598(55)	151/255(59)	248/412(60)	162/262(62)	
Income					<0.001
High	252/598(42)	119/255(47)	178/412(43)	83/262(32)	
Low	49/598(8.2)	15/255(5.9)	44/412(11)	20/262(7.6)	
High average	182/598(30)	69/255(27)	136/412(33)	90/262(34)	
Low average	115/598(19)	52/255(20)	54/412(13)	69/262(26)	
Civil					<0.001
With partner	425/598(71)	205/255(80)	326/412(79)	160/262(61)	
No partner	173/598(29)	50/255(20)	86/412(21)	102/262(39)	

Continue...

Continuation.

Characteristic	BMI				p-value#
	Adequate* n = 598	Obese* n = 255	Overweight* n = 412	Underweight* n = 262	
Region					0.9
Northeast	279/598(47)	115/255(45)	199/412(48)	124/262(47)	
South/Southeast	319/598(53)	140/255(55)	213/412(52)	138/262(53)	
GDM					<0.001
No	557/595(94)	218/253(86)	357/408(88)	250/260(96)	
Yes	38/595(6.4)	35/253(14)	51/408(13)	10/260(3.8)	
Unknown	3	2	4	2	
PE					<0.001
No	560/595(94)	220/253(87)	379/408(93)	248/260(95)	
Yes	35/595(5.9)	33/253(13)	29/408(7.1)	12/260(4.6)	
Unknown	3	2	4	2	
Preterm birth					0.7
No	529/595(89)	223/253(88)	370/408(91)	231/260(89)	
Yes	66/595(11)	30/253(12)	38/408(9.3)	29/260(11)	
Unknown	3	2	4	2	
Spontaneous					0.5
No	547/591(93)	235/247(95)	380/405(94)	237/257(92)	
Yes	44/591(7.4)	12/247(4.9)	25/405(6.2)	20/257(7.8)	
Unknown	7	8	7	5	
Therapeutic					0.071
No	560/582(96)	233/251(93)	390/403(97)	244/253(96)	
Yes	22/582(3.8)	18/251(7.2)	13/403(3.2)	9/253(3.6)	
Unknown	16	4	9	9	
SGA					0.2
No	55/595(9.2)	34/253(13)	36/408(8.8)	30/260(12)	
Yes	540/595(91)	219/253(87)	372/408(91)	230/260(88)	
Unknown	3	2	4	2	
Parturition					<0.001
Elective cesarean section	123/595(21)	85/253(34)	116/408(28)	29/260(11)	
Non-elective cesarean section	140/595(24)	70/253(28)	95/408(23)	46/260(18)	
Vaginal Delivery	312/595(52)	95/253(38)	187/408(46)	181/260(70)	
Surgical Delivery	20/595(3.4)	3/253(1.2)	10/408(2.5)	4/260(1.5)	
Unknown	3	2	4	2	
Apgar 7					0.5
No	564/571(99)	234/239(98)	385/394(98)	248/251(99)	
Yes	7/571(1.2)	5/239(2.1)	9/394(2.3)	3/251(1.2)	
Unknown	27	16	18	11	
Weight_Newborn	3,116.3(583.5)	3,196.1(654.9)	3,182.5(569.2)	3,034.9(524.6)	<0.001
Unknown	16	8	9	12	
Length_Newborn	48.1(3.2)	48.2(3.6)	48.3(3.1)	47.6(3.2)	0.003
Unknown	44	24	27	24	
BMI_2v					<0.001
Adequate	410/503(82)	1/217(0.5)	14/338(4.1)	55/215(26)	
Obese	2/503(0.4)	205/217(94)	40/338(12)	0/215(0)	
Overweight	75/503(15)	10/217(4.6)	284/338(84)	0/215(0)	
Underweight	16/503(3.2)	1/217(0.5)	0/338(0)	160/215(74)	
Unknown	95	38	74	47	
BMI_3v					<0.001
Adequate	285/434(66)	0/171(0)	7/288(2.4)	74/172(43)	
Obese	9/434(2.1)	163/171(95)	75/288(26)	0/172(0)	
Overweight	117/434(27)	8/171(4.7)	206/288(72)	2/172(1.2)	
Underweight	23/434(5.3)	0/171(0)	0/288(0)	96/172(56)	
Unknown	164	84	124	90	

*Mean (SD); n / n(%)

#Kruskal-Wallis rank sum test; Pearson's Chi-squared test; Fisher's exact test

Table 6S. Trajectory of the sum of skinfolds by pregnancy outcomes – Gestational Diabetes Mellitus

Characteristic	Gestational Diabetes Mellitus		p-value#
	No* n = 1,382	Yes* n = 134	
Skinfold Subscapular 1st visit	20.7(8.4)	25.5(10.1)	<0.001
Skinfold Subscapular 2nd visit	22.1(8.1)	26.3(9.6)	<0.001
Unknown	231	17	
Skinfold Subscapular 3rd visit	22.9(8.3)	25.9(10.3)	0.016
Unknown	418	41	
Skinfold Triceps 1st visit	21.2(7.6)	23.8(8.8)	0.002
Skinfold Triceps 2nd visit	22.0(7.3)	23.8(8.7)	0.084
Unknown	230	17	
Skinfold Triceps 3rd visit	22.4(7.6)	23.4(8.8)	0.5
Unknown	415	41	
Skinfold Biceps 1st visit	14.8(6.7)	17.4(8.2)	<0.001
Skinfold Biceps 2nd visit	15.5(6.8)	17.8(7.9)	0.004
Unknown	230	17	
Skinfold Biceps 3rd visit	16.1(6.9)	17.9(7.8)	0.049
Unknown	415	41	
Sum of the 3 Skinfolds 1st visit	72.6(28.0)	84.7(35.9)	<0.001
Sum of the 3 Skinfolds 2nd visit	75.8(27.6)	85.4(35.0)	0.011
Unknown	230	17	
Sum of the 3 Skinfolds 3rd visit	77.4(27.7)	84.9(36.2)	0.14
Unknown	415	41	
Age			<0.001
≤19 Years	347/1,382(25)	17/134(13)	
≥36 Years	54/1,382(3.9)	12/134(9.0)	
20-35 Years	981/1,382(71)	105/134(78)	
Schooling			0.10
>12 Years	402/1,382(29)	48/134(36)	
≤8 Years	175/1,382(13)	10/134(7.5)	
9-12 Years	805/1,382(58)	76/134(57)	
Income			0.016
High	566/1,382(41)	62/134(46)	
Low	108/1,382(7.8)	19/134(14)	
High Average	436/1,382(32)	35/134(26)	
Low Average	272/1,382(20)	18/134(13)	
Civil			0.4
With partner	1,007/1,382(73)	102/134(76)	
No partner	375/1,382(27)	32/134(24)	
Region			<0.001
Northeast	670/1,382(48)	43/134(32)	
South/Southeast	712/1,382(52)	91/134(68)	
GDM			0.9
No	1,283/1,382(93)	124/134(93)	
Yes	99/1,382(7.2)	10/134(7.5)	
PE			0.4
No	1,236/1,382(89)	117/134(87)	
Yes	146/1,382(11)	17/134(13)	
SGA			0.3
No	1,237/1,382(90)	124/134(93)	
Yes	145/1,382(10)	10/134(7.5)	
Preterm Birth			>0.9
No	1,279/1,371(93)	120/129(93)	
Yes	92/1,371(6.7)	9/129(7.0)	
Spontaneous			>0.9
No	1,279/1,371(93)	120/129(93)	
Yes	92/1,371(6.7)	9/129(7.0)	
Unknown	11	5	
Therapeutic			0.3
No	1,301/1,355(96)	126/134(94)	
Yes	54/1,355(4.0)	8/134(6.0)	
Unknown	27	0	

Continue...

Continuation.

Characteristic	Gestational Diabetes Mellitus No* n = 1,382	Gestational Diabetes Mellitus Yes* n = 134	p-value#
Parturition			0.011
Elective cesarean section	311/1,382(23)	42/134(31)	
Non-elective cesarean section	314/1,382(23)	37/134(28)	
Vaginal Delivery	724/1,382(52)	51/134(38)	
Surgical Delivery	33/1,382(2.4)	4/134(3.0)	
Apgar 7			>0.9
No	1,301/1,323(98)	130/132(98)	
Yes	22/1,323(1.7)	2/132(1.5)	
Unknown	59	2	
Weight_Newborn	3,129.2(586.3)	3,181.3(570.6)	0.6
Unknown	33	1	
Length _RN	48.1(3.2)	47.9(3.4)	0.5
Unknown	99	9	

*Mean (SD); n / n(%)
#Kruskal-Wallis rank sum test; Pearson's Chi-squared test; Fisher's exact test

Table 7S. Trajectory of the sum of skinfolds by pregnancy outcomes – Preeclampsia

**Characteristic**	**Preeclampsia No* n = 1,407**	**Preeclampsia Yes* n = 109**	**p-value#**
Skinfold Subscapular 1st visit	20.9(8.5)	24.8(10.6)	<0.001
Skinfold Subscapular 2nd visit	22.2(8.2)	25.8(9.6)	0.003
Unknown	225	23	
Skinfold Subscapular 3rd visit	23.0(8.5)	26.5(9.3)	0.004
Unknown	408	51	
Skinfold Triceps 1st visit	21.3(7.7)	23.7(8.5)	0.007
Skinfold Triceps 2nd visit	22.0(7.4)	24.5(8.5)	0.020
Unknown	224	23	
Skinfold triceps 3rd visit	22.3(7.6)	25.4(9.2)	0.027
Unknown	405	51	
Skinfold biceps 1st visit	14.7(6.7)	18.5(8.4)	<0.001
Skinfold biceps 2nd visit	15.5(6.8)	18.1(8.4)	0.013
Unknown	224	23	
Skinfold biceps 3rd visit	16.0(6.8)	19.1(8.5)	0.008
Unknown	405	51	
Sum of the 3 skinfolds 1st visit	72.7(28.4)	85.2(34.2)	<0.001
Sum of the 3 skinfolds 2nd visit	76.0(28.0)	86.1(33.2)	0.010
Unknown	224	23	
Sum of the 3 skinfolds 3rd visit	77.4(28.3)	89.6(31.1)	0.005
Unknown	405	51	
Age			0.13
≤19 Years	339/1,407(24)	25/109(23)	
≥36 Years	57/1,407(4.1)	9/109(8.3)	
20-35 Years	1,011/1,407(72)	75/109(69)	
Schooling			0.7
>12 Years	420/1,407(30)	30/109(28)	
≤8 Years	169/1,407(12)	16/109(15)	
9-12 Years	818/1,407(58)	63/109(58)	
Income			0.9
High	585/1,407(42)	43/109(39)	
Low	117/1,407(8.3)	10/109(9.2)	
High average	434/1,407(31)	37/109(34)	
Low average	271/1,407(19)	19/109(17)	
Civil			0.9
With partner	1,030/1,407(73)	79/109(72)	
No partner	377/1,407(27)	30/109(28)	
Region			0.12
Northeast	654/1,407(46)	59/109(54)	
South/Southeast	753/1,407(54)	50/109(46)	

Continue...

Continuation.

Characteristic	Preeclampsia No* n = 1,407	Preeclampsia Yes* n = 109	p-value#
GDM			0.9
No	1,283/1,407(91)	99/109(91)	
Yes	124/1,407(8.8)	10/109(9.2)	
SGA			<0.001
No	1,276/1,407(91)	85/109(78)	
Yes	131/1,407(9.3)	24/109(22)	
Preterm birth			0.2
No	1,299/1,396(93)	100/104(96)	
Yes	97/1,396(6.9)	4/104(3.8)	
Spontaneous			0.2
No	1,299/1,396(93)	100/104(96)	
Yes	97/1,396(6.9)	4/104(3.8)	
Unknown	11	5	
Therapeutic			<0.001
No	1,342/1,382(97)	85/107(79)	
Yes	40/1,382(2.9)	22/107(21)	
Unknown	25	2	
Parturition			<0.001
Elective cesarean section	304/1,407(22)	49/109(45)	
Non-elective cesarean section	320/1,407(23)	31/109(28)	
Vaginal delivery	748/1,407(53)	27/109(25)	
Surgical delivery	35/1,407(2.5)	2/109(1.8)	
Apgar 7			0.4
No	1,328/1,349(98)	103/106(97)	
Yes	21/1,349(1.6)	3/106(2.8)	
Unknown	58	3	
Weight_Newborn	3,161.0(546.4)	2,782.3(883.6)	<0.001
Unknown	31	3	
Length _Newborn	48.2(3.1)	46.8(4.8)	0.002
Unknown	98	10	

*Mean (SD); n / n(%)
#Wilcoxon rank sum test; Fisher's exact test; Pearson's Chi-squared test

Table 8S. Trajectory of the sum of skinfolds by pregnancy outcomes – Preterm birth

Characteristic	Preterm birth No* n = 1,353	Preterm birth Yes* n = 163	p-value#
Skinfold subscapular 1st visit	21.2(8.7)	21.0(8.6)	>0.9
Skinfold subscapular 2nd visit	22.6(8.4)	21.7(8.0)	0.3
Unknown	204	44	
Skinfold triceps 1st visit	21.6(7.8)	20.8(7.3)	0.3
Skinfold triceps 2nd visit	22.4(7.5)	20.6(7.3)	0.020
Unknown	203	44	
Skinfold biceps 1st visit	15.0(6.9)	15.2(6.8)	0.8
Skinfold biceps 2nd visit	15.7(6.9)	15.5(7.2)	0.6
Unknown	203	44	
Sum of the 3 skinfolds 1st visit	73.7(29.0)	73.3(29.2)	0.9
Sum of the 3 skinfolds 2nd visit	77.0(28.4)	74.0(29.1)	0.3
Unknown	203	44	
Age			0.2
≤19 Years	333/1,353(25)	31/163(19)	
≥36 Years	56/1,353(4.1)	10/163(6.1)	
20-35 Years	964/1,353(71)	122/163(75)	
Schooling			0.7
>12 Years	403/1,353(30)	47/163(29)	
≤8 Years	168/1,353(12)	17/163(10)	
9-12 Years	782/1,353(58)	99/163(61)	
Income			0.7
High	560/1,353(41)	68/163(42)	
Low	115/1,353(8.5)	12/163(7.4)	
High average	415/1,353(31)	56/163(34)	
Media baixa	263/1,353(19)	27/163(17)	

Continue...

Continuation.

Characteristic	Preterm birth No* n = 1,353	Preterm birth Yes* n = 163	p-value#
Civil			0.5
With partner	993/1,353(73)	116/163(71)	
No partner	360/1,353(27)	47/163(29)	
Region			0.2
Northeast	644/1,353(48)	69/163(42)	
South/Southeast	709/1,353(52)	94/163(58)	
DMG			0.4
No	1,236/1,353(91)	146/163(90)	
Yes	117/1,353(8.6)	17/163(10)	
PE			<0.001
No	1,270/1,353(94)	137/163(84)	
Yes	83/1,353(6.1)	26/163(16)	
SGA			<0.001
No	1,232/1,353(91)	129/163(79)	
Yes	121/1,353(8.9)	34/163(21)	
Spontaneous			<0.001
No	1,353/1,353(100)	46/147(31)	
Yes	0/1,353(0)	101/147(69)	
Unknown	0	16	
Therapeutic			<0.001
No	1,353/1,353(100)	74/136(54)	
Yes	0/1,353(0)	62/136(46)	
Unknown	0	27	
Income			<0.001
High	292/1,353(22)	61/163(37)	
Low	331/1,353(24)	20/163(12)	
High average	694/1,353(51)	81/163(50)	
Low average	36/1,353(2.7)	1/163(0.6)	
Apgar 7			<0.001
No	1,288/1,298(99)	143/157(91)	
Yes	10/1,298(0.8)	14/157(8.9)	
Unknown	55	6	
Weight_Newborn	3,257.3(418.4)	2,114.1(747.5)	<0.001
Unknown	31	3	
Length _Newborn	48.7(2.3)	43.0(5.2)	<0.001
Unknown	89	19	

*Mean (SD); n / n(%)

#Wilcoxon rank sum test; Pearson's Chi-squared test; Fisher's exact test

11 missing observations have been removed

Table9S. Trajectory of the sum of skinfolds by pregnancy outcomes – Small for gestational age

Characteristic	SGA No* n = 1361	SGA Yes* n = 155	p-value#
Skinfold subscapular 1st visit	21.0(8.7)	22.0(8.9)	0.2
Skinfold subscapular 2nd visit	22.4(8.3)	23.1(8.7)	0.4
Unknown	216	32	
Skinfold subscapular 3rd visit	23.1(8.5)	23.9(9.2)	0.4
Unknown	397	62	
Skinfold triceps 1st visit	21.4(7.7)	22.2(8.1)	0.2
Skinfold triceps 2nd visit	22.1(7.4)	23.0(8.2)	0.3
Unknown	215	32	
Skinfold triceps 3rd visit	22.4(7.7)	23.5(7.5)	
Unknown	395	61	
Skinfold biceps 1st visit	14.9(6.8)	15.7(7.6)	0.3
Skinfold biceps 2nd visit	15.7(6.9)	15.8(7.5)	0.8
Unknown	215	32	
Skinfold biceps 3rd visit	16.1(6.9)	17.2(7.6)	0.2
Unknown	395	61	
Sum of the 3 skinfolds 1st visit	73.0(28.7)	79.6(30.8)	0.010
Sum of the 3 skinfolds 2nd visit	76.2(28.3)	80.7(30.1)	0.10

Continue...

Continuation.

Characteristic	SGA		p-value#
	No* n = 1361	Yes* n = 155	
Unknown	215	32	
Sum of the 3 skinfolds 3rd visit	77.4(28.5)	85.0(29.2)	0.014
Unknown	395	61	
Age			0.7
≤19 Years	329/1,361(24)	35/155(23)	
≥36 Years	61/1,361(4.5)	5/155(3.2)	
20-35 Years	971/1,361(71)	115/155(74)	
Schooling			0.4
>12 Years	408/1,361(30)	42/155(27)	
≤8 Years	170/1,361(12)	15/155(9.7)	
9-12 Years	783/1,361(58)	98/155(63)	
Income			0.036
High	573/1,361(42)	55/155(35)	
Low	115/1,361(8.4)	12/155(7.7)	
High average	407/1,361(30)	64/155(41)	
Low average	266/1,361(20)	24/155(15)	
Civil			0.8
With partner	997/1,361(73)	112/155(72)	
No partner	364/1,361(27)	43/155(28)	
Region			0.6
Northeast	637/1,361(47)	76/155(49)	
South/Southeast	724/1,361(53)	79/155(51)	
GDM			0.3
No	1,237/1,361(91)	145/155(94)	
Yes	124/1,361(9.1)	10/155(6.5)	
PE			<0.001
No	1,276/1,361(94)	131/155(85)	
Yes	85/1,361(6.2)	24/155(15)	
Preterm Birth			<0.001
No	1,232/1,361(91)	121/155(78)	
Yes	129/1,361(9.5)	34/155(22)	
Spontaneous			0.7
No	1,258/1,350(93)	141/150(94)	
Yes	92/1,350(6.8)	9/150(6.0)	
Unknown	11	5	
Therapeutic			<0.001
No	1,298/1,335(97)	129/154(84)	
Yes	37/1,335(2.8)	25/154(16)	
Unknown	26	1	
Parturition			<0.001
Elective cesarean section	304/1,361(22)	49/155(32)	
Non-elective cesarean section	332/1,361(24)	19/155(12)	
Vaginal delivery	690/1,361(51)	85/155(55)	
Surgical delivery	35/1,361(2.6)	2/155(1.3)	
Apgar 7			0.5
No	1,283/1,306(98)	148/149(99)	
Yes	23/1,306(1.8)	1/149(0.7)	
Unknown	55	6	
Weight _RN	3,222.7(506.8)	2,357.0(646.1)	<0.001
Unknown	31	3	
Length _ Newborn	48.4(2.9)	45.3(4.4)	<0.001
Unknown	97	11	

*Mean (SD); n / n(%)

#Wilcoxon rank sum test; Fisher's exact test; Pearson's Chi-squared test
